# Multi-Omics Data Integration for Improved Cancer Subtyping via Denoising Autoencoder-Based Multi-Kernel Learning

**DOI:** 10.3390/genes16111246

**Published:** 2025-10-22

**Authors:** Xiukun Yao, Tong Wang, Qi Yang, Jiawen Wang, Yao Qi, Tong Xu, Zhiwen Wei, Yuehua Cui, Hongyan Cao, Keming Yun

**Affiliations:** 1 Academy of Forensic Medicine, Shanxi Medical University, Jinzhong 030600, China; yxksy@sxmu.edu.cn (X.Y.); w2041223@163.com (J.W.); 19854195522@163.com (Y.Q.); weizhiwen2000@163.com (Z.W.); 2 Key Laboratory of Forensic Medicine in Shanxi Province, Jinzhong 030600, China; 3 Key Laboratory of Forensic Toxicology, Ministry of Public Security, Jinzhong 030600, China; 4 Shanxi Provincial Key Laboratory of Major Diseases Risk Assessment, Department of Health Statistics, School of Public Health, Shanxi Medical University, Jinzhong 030600, China; wangtong91@sxmu.edu.cn (T.W.); m18226200197@163.com (Q.Y.); xutong20201@163.com (T.X.); caohy@sxmu.edu.cn (H.C.); 5 MOE Key Laboratory of Coal Environmental Pathogenicity and Prevention, Shanxi Medical University, Jinzhong 030600, China; 6 Department of Statistics and Probability, Michigan State University, East Lansing, MI 48824, USA; cuiy@msu.edu

**Keywords:** multi-omics data integration, subtypes identification, denoising autoencoder, hierarchical multi-kernel learning, deep learning

## Abstract

**Objectives**: Cancer, characterized by its profound complexity and heterogeneity, arises from a multitude of molecular disruptions. The pursuit of identifying distinct cancer subtypes is driven by the need to stratify patients into clinically coherent subgroups, each exhibiting unique prognostic outcomes. The integration of multi-omics datasets enhances the precision of subtyping and advances precision medicine. **Methods**: Considering the high-dimensional nature inherent to various multi-omics data types, we introduce an innovative deep learning framework, DAE-MKL, which integrates denoising autoencoders with multi-kernel learning for identifying cancer subtypes. Leveraging the capabilities of DAE, we extract non-linearly transformed features that retain pertinent information while mitigating noise and redundancy. These refined data representations are then funneled into the MKL framework, thereby enhancing the accuracy of subtype identification. We applied the DAE-MKL framework to both simulated studies and empirical datasets derived from two distinct cancer types, low-grade glioma (LGG, n = 86) and kidney renal clear cell carcinoma (KIRC, n = 285), thereby validating its utility and feasibility. **Results**: In simulations, DAE-MKL achieved superior performance with NMI gains up to 0.78 compared to other state-of-the-art methods. For real datasets, DAE-MKL identified three LGG subtypes and three KIRC subtypes, showing significant survival differences (KIRC log-rank p = 3.33 × 10^−8^, LGG log-rank p = 3.99 × 10^−8^). Additionally, we explored potential cancer-related biomarkers. **Conclusions**: The DAE-MKL effectively identifies molecular subtypes, reduces data dimensionality, and improves prognostic stratification in multi-omics cancer datasets, providing an effective tool for precision oncology.

## 1. Introduction

Tumor heterogeneity is one of the major reasons that fail the traditional histopathology [1,2]. Patients with the same cancer type may exhibit distinct clinical trajectories due to different molecular alterations [3]. Establishing tumor molecular subtypes based on these distinct molecular biological characteristics and conducting personalized treatment is an important development direction in clinical oncology [4].

The molecular mechanisms of tumors are highly complex, and their occurrence and development involve intricate regulatory mechanisms of multiple omics. With the rapid advancement of high-throughput sequencing technologies, expansive multi-omics datasets are unveiling various molecular insights [5,6], thereby aiding in the elucidation of underlying pathogenic mechanisms and providing unprecedented opportunities for advancing precision medicine strategies. Integrating data from various omics not only enhances our understanding of biological processes but also enables the identification of molecular subtypes [7]. Computational methods have been developed to facilitate multi-omics integration, which can be divided into three categories based on different fusion strategies [8,9]. Early integration methods, such as LRAcluster [10], concatenate each dataset into a large input matrix, upon which any single-omic clustering algorithm can be applied for analysis. However, this approach assumes that each data type contributes equally, potentially leading to information loss and bias. Additionally, it results in increased dimensionality when data are directly merged [11]. In contrast, late integration methods, such as COCA [12], apply single-omic clustering algorithms to each dataset separately and then integrate the different clustering results. While applicable to any clustering algorithm, this method is prone to losing signals that are weak in each omic separately. The third category is mixed integration, which mitigates the limitations of early integration by mapping each omics dataset into a lower-dimensional, less noisy latent space. This transformation reduces heterogeneity arising from data type and scale, enabling the combined representation to be analyzed by standard ML models. Representative methods within this category include kernel-based methods such as SNF [13] and CIMLR [14]. Notably, CIMLR learns the weights of multiple kernels within each data type, while simultaneously optimizing kernel parameters across all omics. However, this optimization may overlook different omics heterogeneity. Wei et al. proposed a novel kernel-based method, termed hierarchical multi-kernel learning (hMKL) [15], to address the limitations of CIMLR. The hMKL captures the heterogeneity across different omics data by constructing a composite kernel for each data type, which are then integrated into a unified fused kernel. This approach facilitates the identification of distinct cancer subtypes and their biomarkers, highlighting its potential for multi-omics data integration. Nevertheless, a key limitation of hMKL lies in the kernel construction process, where measurement constraints and intrinsic biological variability inevitably introduce noisy features. Such noise can dilute subtyping signals and weakens the learning capability. Therefore, it is critical to implement strategies that reduce noise and dimensionality, while preserving the underlying subtype-relevant information embedded in multi-omics data.

In recent years, deep learning has emerged as a powerful approach for handling high-dimensional data, owing to its capacity to learn nonlinear and complex feature representations that enhance clustering performance. Among these approaches, autoencoders have been extensively employed for feature extraction [16]. They map inputs to outputs through an encoder-decoder architecture and provide unique advantages in denoising and dimensionality reduction. By capturing the global structure of high-dimensional features while reducing redundancy. Building on the autoencoders, the Denoising Autoencoder (DAE) introduces noise into the input during training, compelling the model to recover the original signal. This design prevents the network from learning a trivial identity mapping and yields more robust latent feature representations [17,18]. Given the advantages of DAE, it is crucial to develop novel frameworks for unsupervised kernel fusion by incorporating deep-learning dimension reduction. Therefore, we propose a novel deep learning framework DAE-MKL, which employs hMKL to construct a fused sample similarity matrix by integrating the refined representations extracted from the DAE method. This fused similarity matrix strengthens true similarities, suppresses spurious associations, and reduces noise, thereby improving the accuracy of subtype identification.

We performed simulation studies to assess the effectiveness and robustness of the proposed DAE-MKL method with the original hMKL method under different conditions. We further applied the DAE-MKL method to two cancer types, low-grade glioma (LGG) and kidney renal clear cell carcinoma (KIRC), obtained from Chinese Glioma Genome Atlas (CGGA) [6] and Cancer Genome Atlas (TCGA) [5], respectively, to demonstrate their utility and practicability. The results showed that the DAE-MKL method outperforms the hMKL method and other state-of-the-art methods in both simulation studies and practical applications. The molecular subtypes identified in LGG and KIRC patients, along with subsequent biological analysis of critical molecular characteristics and pathways, provide novel perspectives for improving the personalized precision treatment of tumor diseases.

## 2. Materials and Methods

### 2.1. DAE-MKL

**Figure 1:**
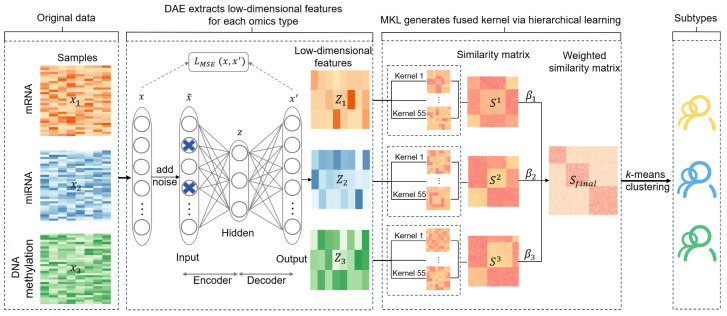
The workflow of DAE-MKL. The process begins with dimension reduction for each omics data type, including miRNA, mRNA, and DNA methylation. A hierarchical kernel learning strategy hMKL is then employed to the refined representations, which first learns a composite kernel by optimizing the kernel parameters and kernel weight for each data type, and then integrates all composite kernels into a final kernel under the UMKL framework. Finally, k-means clustering is applied to the fused kernel to identify cancer subtypes.

DAE-MKL incorporates a denoising autoencoder within the MKL framework to perform deep-learning dimension-reduction, reconstructing features from multi-omics data to improve subtype identification. It consists of three main steps (See Figure 1): (1) Deep-learning dimension reduction for each omics data type. DAE is applied to perform nonlinear transformation and feature learning, enabling the extraction of a more robust latent representation for features. (2) Hierarchical multi-kernel learning. The reconstructed features from step (1) serve as input for multi-kernel learning to optimize kernel parameters, where kernel parameters and kernel weight are optimized to learn a composite kernel. These individual kernels are then fused into a final kernel through a weighted linear combination using an unsupervised multiple kernel learning method (UMKL). (3) Cancer subtyping. K-means clustering is performed on the final fused kernel to obtain sample clusters for cancer subtyping.

**Stage 1**: Extracting low-dimensional representations of each data type using DAE. We first utilize DAE to generate a low-dimensional representation for each omics data type, such as mRNA, miRNA, and DNA methylation. Suppose we have M types of omics data for n patients, which denoted as $x_{m}\;(m=1,\;\cdots ,\;M)$. For each omic data type, given input data x, DAE trains the input features with added noise to prevent the network from learning an identity mapping, which would be pointless. This strategy enables the model to better extract meaningful features from noisy data. Common types of noise include Gaussian noise and random dropout:

(1) $$\tilde{x}=x+N(0,\sigma^{2})\;\mathrm{or}\;\tilde{x}=mask(x)$$

where $N(0,\sigma^{2})$ represents Gaussian noise zero mean and a variance $\sigma^{2}$, mask(·) randomly drops part of the input. In our study, we adopted random dropout as the noise type for all omics datasets. The noise data $\tilde{x}$ are then used as the input to the encoder. The encoder formula of the autoencoder is:

(2) $$z=f_{encoder}(\tilde{x})$$

The hidden layer represents a low-dimensional mapping of the noisy data by the encoder, and then the decoder reconstructs the input data to closely match the original data. This process aims to learn key input features, and the decoder formula is:

(3) $$x^{\prime }=f_{decoder}\left(z\right)$$

Here, the DAE uses the ReLU activation function, defined as $f(\tilde{x})=max(0,\tilde{x})$, which is applied to all layers in the encoder and the intermediate layers of the decoder. The goal is to minimize reconstruction error, ensuring that the decoder’s output closely matches the original data. The loss function is introduced to minimize the error between x and $x^{\prime }$, as follows:

(4) $$L_{MSE}={∥x-x^{\prime }∥}_{2}^{2}={∥x-f_{decoder}(f_{decoder}(\tilde{x}))∥}_{2}^{2}$$

When the loss function is minimized and the number of hidden layer nodes is less than the input layer nodes, the optimal latent feature representation (z) is obtained. L1 and L2 regularization are then added to enhance the generalization ability and robustness of the autoencoder. After repeated training, the optimal parameters are determined, with the optimal number of hidden layer nodes selected through grid search based on data analysis [19,20]. In this study, final hyperparameters, derived from extensive experiments, are as follows: the input and hidden dropout ratios are set to 0.1 and 0.3, respectively, and L1 and L2 regularization values are set to 0.006 and 0.1. A sensitivity analysis exploring different dropout combinations was performed using simulated data (variance = 12, signal-to-noise ratio = 10%), with detailed results presented in Supplementary Note S1 (Table S1 and Figure S1). The analysis indicates that optimal clustering performance is achieved with input dropout 0.1–0.3 and hidden dropout 0.3–0.5. The resulting low-dimensional feature matrices are denoted as ${Ζ}_{m}\;(m=1,\;\cdots ,\;M)$, with dimension $n\times p_{m}$.

**Stage 2**: Obtain the fused kernel under the hMKL framework. Given the refined representations ${Ζ}_{m}$ for each data type from stage 1, a hierarchical kernel learning strategy, hMKL, is employed, which first learns a composite kernel by optimizing the kernel parameters and weights for each data type, and then integrates all composite kernels into a final kernel under the UMKL framework.

Step 1. Construct Gaussian kernels and optimize their parameters and weights within the CIMLR framework to learn sample–sample similarities for each omics data type.

The Gaussian kernel [13] function is defined as follows:

(5) $$K\left(z_{i},z_{j}\right)=\frac{1}{\varepsilon_{ij}\sqrt{2\pi }}exp(-\frac{{\left\|z_{i}-z_{j}\right\|}_{2}^{2}}{2\varepsilon_{ij}^{2}})$$

where ${\left\|z_{i}-z_{j}\right\|}_{2}$ is the Euclidean distance between sample i and j. The variance can be calculated as:

(6) $$\mu_{i}=\frac{\sum_{l\in KNN(z_{i}){\left\|z_{i}-z_{j}\right\|}_{2}}}{k},\varepsilon_{ij}=\frac{\sigma (\mu_{i}+\mu_{j})}{2}$$

where $KNN(z_{i})$ represents samples that are top k neighbors of the samples i. A total of 55 Gaussian kernels can be can be constructed for each data type by varying the parameters $\left(\sigma ,k\right)$, where $\sigma \in \left[1,2\right]$ with a step size of 0.25, and $k\in \left[10,30\right]$ with a step size of 2 (See Ramazzotti et al. [14] for details). The distance and kernel between samples i and j can generally be represented as: $D\left(i,j\right)=2-2\sum_{l}w_{l}K_{l}(z_{i},z_{j})$, where $w_{l}$ represents the weight of each kernel K(.,.). $w_{l}$ represents the importance of each individual kernel $K_{l}$.

We compute the pairwise similarities between samples across multiple data types through the following optimization framework under the CIMLR method:

(7) $$\underset{S,L,w}{minimize}-\sum_{i,j,l}w_{l}K_{l}\left(z_{i},z_{j}\right)S_{ij}+\beta {\left\|S\right\|}_{F}^{2}+\gamma tr\left(L^{T}\left(I_{n}-S\right)L\right)+\rho \sum_{l}w_{l}\log w_{l}\;subject\;to\;L^{T}L=I_{c},\sum_{l}w_{l}=1,w_{l}\geq 0,\sum_{j}S_{ij}=1,\;and\;S_{ij}\geq 0$$

where tr represents the matrix trace, and β and γ are two non-negative tuning parameters, $I_{n}$ and $I_{c}$ denote the identity matrices of size n×n and C×C, respectively, with C is the predefined number of classes, ${\left\|S\right\|}_{F}$ denotes the Frobenius norm of the similarity matrix S, and L is a low-dimensional matrix that imposes a low-rank structure on S. The optimization objective involves solving three variables: the similarity matrix S, the weight vector w, and the low-rank constraint matrix L. The final similarity matrix S for each data type is then obtained.

Step 2. Obtain the final-weighted similarity matrices within the UMKL framework.

Based on the similarity matrix S of each data type from step 1, UMKL first constructs a *k*-nearest neighbor graph G, which is associated with each kernel. Then, an n×n matrix Q that represents the original topological structure of the data, where $Q_{ij}$ indicates the frequency with which the pair $\left(i,j\right)$ appears in the edge list of G. Specifically, an N-dimensional vector ${∆}_{i}\left(\beta \right)$ is introduced, defined as $\;{∆}_{i}\left(\beta \right)=\left(\begin{array}{c} S_{i1}^{*}\\ \vdots \\ S_{in}^{*} \end{array}\right)$, where $\left(S_{i1}^{*}\cdots S_{in}^{*}\right)$ represent the similarities between sample i and the other samples. UMKL directly uses the kernel matrix S to measure the topological structure between samples, rather than using distances in the original data space. The optimization problem is as follows:

(8) $$\underset{\beta }{\min}\mathrm{mize}\sum_{i,j=1}^{n}Q_{ij}{\left\|{∆}_{i}\left(\beta \right)-{∆}_{j}\left(\beta \right)\right\|}^{2}\;for\;S^{*}=\sum_{m=1}^{M}\beta_{m}S^{m}\;\beta \in R^{M}\;such\;that\;\beta_{m}\geq 0\;and\;\sum_{m=1}^{M}\beta_{m}=1$$

where $S^{m}\left(m=1,\cdots ,M\right)$ is the mth similarity matrix, M is the total number of similarity matrix. Equation (8) can be rewritten as:

(9) $$\underset{\beta }{\mathrm{minmize}}\sum_{m,m^{\prime }=1}^{M}\beta_{m}\beta_{m^{\prime }}S^{{\mathrm{mm}}^{\prime }}\;for\;\beta \in R^{M}\;such\;that\;\beta_{m}\geq 0\;and\;\sum_{m=1}^{M}\beta_{m}=1\;for\;S^{{\mathrm{mm}}^{\prime }}=\sum_{i,j=1}^{n}S_{ij}\langle {∆}_{i}^{m}-{∆}_{j}^{m},{∆}_{i}^{m^{\prime }}-{∆}_{j}^{m^{\prime }}\rangle \;and\;{∆}_{i}^{m}=\left(\begin{array}{c} S_{i1}^{m}\\ \vdots \\ S_{in}^{m} \end{array}\right)$$

where $S^{mm^{\prime }}$ can be obtained by solving the Quadratic Programming problem in Equation (9) and $\beta_{m}$ for each omics is derived by solving the $L_{1}$ constraint in the QP problem. This allows for the obtained of the weights for each omics similarity matrix, representing the relative contribution of each omics. The final fused kernel matrix is expressed as:

(10) $$S_{final}=\sum_{m=1}^{M}\beta_{m}S^{m},\beta_{m}\geq 0\;and\;\sum_{m=1}^{M}\beta_{m}=1$$

where $\beta_{m}$ represents the weights of $S^{m}$.

**Stage 3**: Use the *k*-means clustering to identify cancer subtypes. Based on the final fused kernel matrix $S_{final}$, *k*-means clustering method [21] is applied to obtain sample clusters. Assuming *k*-means divides all samples into k clusters, denoted as $C_{1},\cdots C_{k}$, the objective is to minimize the squared error E:

(11) $$E=\sum_{C=1}^{k}\sum_{z\in C_{k}}{\left\|S_{final}-u_{k}\right\|}_{2}^{2}$$

where $u_{k}$ is the mean of $C_{k}$.

### 2.2. Estimating the Optimal Number of Clusters

Accurately estimating the number of clusters plays a pivotal role in cancer subtyping. Separation cost [13] is employed to determine the optimal number of clusters. Given a pre-set number of clusters k, the goal is to find an indicator matrix Z(R)=UR, where U is the matrix of the first k eigenvectors of the similarity Laplacian. Let $[M(R){]}_{i}=\underset{j}{max}\;[Z(R){]}_{i,j}$. The cost function can be expressed as:

(12) $$\xi \left(R\right)=\sum_{i,j}\;\frac{{\left[Z\left(R\right)\right]}_{i,j}^{2}}{{\left[M\left(R\right)\right]}_{i}^{2}}$$

The gradient descent method [13] is used to minimize the objective function, and the value of k that produces the largest drop in ξ(R) is determined the optimal number of clusters.

### 2.3. Simulation Study

We conducted simulation studies to evaluate the performance of the DAE-MKL method for subtype identification using multi-omics data and compared it with several state-of-the-art methods, including SAE-MKL, AE-MKL, hMKL, CIMLR [14] and SNF [13]. Similar to DAE-MKL, the SAE-MKL and AE-MKL approaches are constructed upon sparse autoencoders (SAE) and autoencoders (AE) architectures, respectively, and integrate multi-kernel learning for subtype identification. The simulation design follows the procedures outlined in the literature [20,22,23], with four subtype groups being simulated across three omics data types. The overall clustering structure could only be obtained by integrating information from all omics types, which cannot be achieved at a single omics level. Here, two simulation scenarios were considered. In Scenarios I, we simulated three types of omics data, each consisting of 200 samples and 1000 features. These 200 samples were pre-defined into four subtypes, with each subtype containing 50 samples. Integrating all three omics data is essential for accurately classifying the four subtypes. Considering the independence and overlap among the datasets, three datasets were constructed using the formula $X_{i}^{s}=mean^{s}+\varepsilon$, where $mean^{s}$ represents the mean expression level of features for each dataset, and $\varepsilon ∼N(0,\sigma^{2})$ represents random Gaussian noise. In Scenarios Ⅱ, we combined real data from the Gene Expression Omnibus (GEO) database with predefined cluster structures. The GEO database includes GSE10645 [24] for RNA expression, GSE73002 [25] for miRNA expression and GSE51557 [26] for DNA methylation. Singular value decomposition (SVD) was applied to decompose and reconstruct the actual genomic data with predefined cluster structures. In the two simulation scenarios described above, three levels of noise were set with variances of 4, 8, and 12, respectively, and corresponding signal ratios of 5%, 7.5%, and 10%.

In each scenario, we constructed two datasets: SimData1 and SimData2. SimData1 has a clear boundary between subtypes, whereas SimData2 possesses fuzzy boundaries. SimData2 is based on SimData1, which involves randomly sampling 10–20% of the samples in each subtype.

### 2.4. Simulation Result Evaluation

The Normalized Mutual Information (NMI) metric [27] is widely utilized in the evaluation of the clustering performances of different methods. Given two clustering results A and B, the NMI is defined as:

(13) $$NMI\left(A,B\right)=\frac{I(A,B)}{\sqrt{H(A)H(B)}}$$

where I(A,B) represents the mutual information between A and B, and H(A) and H(B) denote the entropy of clustering results A and B, respectively. The NMI value ranges from 0 to 1, serving as a measure of the consistency between the two clustering results. A higher NMI indicates better alignment with ground truth labels, thus reflecting higher accuracy.

### 2.5. Multi-Omics Data and Data Processing

We focused on subtypes of LOWER-grade gliomas (LGG) and Kidney Renal Clear Cell Carcinoma (KIRC). LGG, classified as World Health Organization (WHO) grades II and III based on their histopathological features, are among the most common infiltrative tumors in the adult cerebral hemispheres. Some of these neoplasms, which are rarely curable, may progress to transform into higher-grade tumors (WHO grade IV, Glioma) [28,29]. Due to the insufficient availability of reliable biomarkers for accurately predicting overall survival in LGG patients, combined with the significant heterogeneity within this tumor group, further molecular subtyping has become increasingly essential. KIRC is one of the most prevalent forms of renal cell carcinoma (RCC), accounting for approximately 70–80% of RCC cases. This subtype is notably aggressive, frequently leading to metastasis and poor prognosis [30]. The heterogeneity among patients makes it difficult to replicate individualized treatment plans. Therefore, conducting extensive research into the molecular mechanisms of KIRC and developing strategies to guide personalized treatment remains essential [31].

The Glioma dataset (including mRNA expression, miRNA expression, DNA methylation, and clinical data) was obtained from the CGGA database, while the KIRC dataset was downloaded from the TCGA website using the TCGAbiolinks software (version 2.36.0) [32]. For LGG, we selected samples classified as pathologic grades II and III to serve as the LGG dataset. The LGG and KIRC datasets were preprocessed as follows:

(1) For LGG datasets, we first selected samples that contained all three omics data, while excluding samples with missing values in clinical factors such as overall survival, survival status, age, gender, and WHO grade, and obtained 86 LGG samples with 827 miRNAs, 19,416 mRNAs, and 14,476 methylation genes. The publicly available multi-omics data were pre-normalized and quality-controlled by the data providers, ensuring consistency and comparability across samples. Next, we performed feature selection based on the most variant Median Absolute Deviation (MAD) to select the top number of value features.

(2) For KIRC datasets, annotated the promoter region CpG methylation sites within 2 kbp of the transcription start site [33], and removed the CpG sites located on sex chromosomes. The remaining CpG sites were mapped to genes, and the mean beta value of multiple CpG sites per gene was used as the gene-level methylation signal; Features with a deletion ratio greater than 30% were removed, and the remaining missing values were imputed using the K-nearest neighbor (KNN) algorithm [34]. Meanwhile miRNA and mRNA data were transformed using a ${log}_{2}(x+1)$ conversion. After these steps, we obtained 285 KIRC samples with 388 miRNAs, 16,893 mRNAs, and 14,296 methylation genes. Subsequently, we performed feature selection based on the most variant Median Absolute Deviation (MAD) to select the top number of value features for each type of omics data.

After preprocessing, we obtained 86 LGG samples with 500 miRNAs, 15,000 mRNAs, and 10,000 methylation genes, as well as 285 KIRC samples with 388 miRNAs, 15,000 mRNAs, and 10,000 methylation genes.

### 2.6. Downstream Statistical Analysis After Subtyping

*Differential* *analysis*: We performed differential expression analysis to explore the molecular heterogeneity and further validate the biological significance of each subtype. The Kruskal–Wallis H test was used to identify differentially expressed miRNAs (DEmiRNAs), differentially expressed mRNAs (DEmRNAs), and differentially methylated genes (DMGs), with a significance threshold set at an FDR-adjusted *p*-value < 0.05. To further assess feature enrichment in each subtype, the hypergeometric distribution test [14] was employed, applying a filtering criterion of $P_{adj}\;$< 0.05. Additionally, miRWalk [35] was utilized to predict the target genes of the identified DEmiRNAs.

*KEGG and GO enrichment analysis:* Gene enrichment analysis was conducted to elucidate the biological processes and pathways represented in the omics data, thereby providing deeper insights into the underlying molecular mechanisms [36]. Well-established methods for enrichment analysis include the Kyoto Encyclopedia of Genes and Genomes (KEGG) [37] and Gene Ontology (GO) [38] analyses. KEGG enrichment analysis focuses on understanding the functional roles of genes and the pathways they are involved in, while GO enrichment analysis categorizes the functions of differentially expressed genes into three main groups: Biological Process (BP), Molecular Function (MF), and Cellular Component (CC). We utilized the R package clusterProfiler (version 4.16.0) [39] to conduct (GO) and (KEGG) enrichment analyses on the overlapping genes across the three omics datasets.

*Immune cell infiltration and Pathway activity analysis:* We employed the R package IOBR (version 0.99.99) [40] to estimate tumor cell composition and identified immune infiltrating cells with significant differences between subtypes using the Kruskal–Wallis H test, with a threshold of $P_{adj}$ < 0.05. Additionally, we characterized differential pathway activities across subtypes by analyzing pathway activity scores for 14 signaling pathways based on gene expression data using the PROGENy package (version 1.30.0) [41].

## 3. Results

### 3.1. Simulation Results

**Figure 2:**
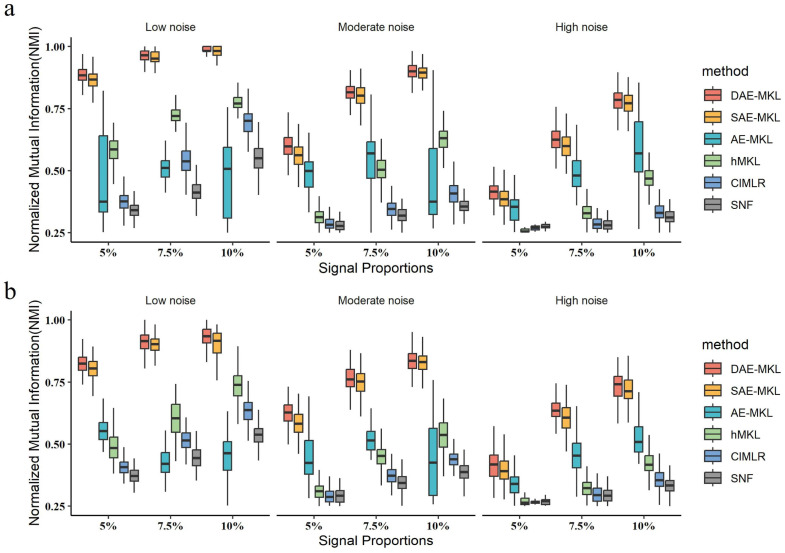
Distribution of NMI values under Scenario I. (**a**) NMI value distribution for SimData1. (**b**) NMI value distribution for SimData2.

The simulation study demonstrated that DAE-MKL outperforms SAE-MKL, AE-MKL, hMKL, CIMLR and SNF across all settings in both two simulation scenarios. Table 1 and Figure 2 show the differences in NMI values among these methods across 1000 replicates under Scenario I. The corresponding results for Scenario II are provided in the Supplementary Materials (see Table S2 and Figure S2). NMI values for all methods increase as the signal ratio increases when the noise ratio is fixed. Notably, the NMI values for the clustering results obtained using DAE-MKL are consistently higher than those of the other methods, indicating that DAE-MKL is more accurate in identifying subtypes and better at capturing the clustering structure of multi-omics data. As shown in Table 1, under a 10% signal strength and high noise setting, the NMI value for DAE-MKL is 0.780, compared to 0.769 for SAE-MKL, 0.468 for hMKL, 0.328 for CIMLR, 0.313 for SNF, and 0.206 for AE-MKL. In SimData2, which simulates fuzzy boundaries, there is a slight overall decrease in NMI, but the trend remains consistent.

### 3.2. Overall Performance of the DAE-MKL Method in LGG and KIRC

We compared the performance of DAE-MKL with other multi-omics integrative subtyping methods in cancer subtyping using the LGG and KIRC datasets, including SAE-MKL, AE-MKL, hMKL, CIMLR, and SNF. The results show that DAE-MKL performs better than other methods, particularly in terms of log-rank p value (See Table 2). Consistent with our findings in the simulation study, DAE-MKL shows better performance across all datasets and exhibits greater distinction in survival rate compared to the other five methods. To enhance the internal validation of our proposed method, we computed several internal clustering validation indices (Connectivity, Silhouette Width, and Dunn Index) for the three identified subtypes in both the KIRC and LGG datasets. Detailed results are presented in Supplementary Note S3 (Table S3), showing that the identified clusters exhibit reasonable compactness and separation.

### 3.3. Subtyping Stability Analysis

**Figure 3:**
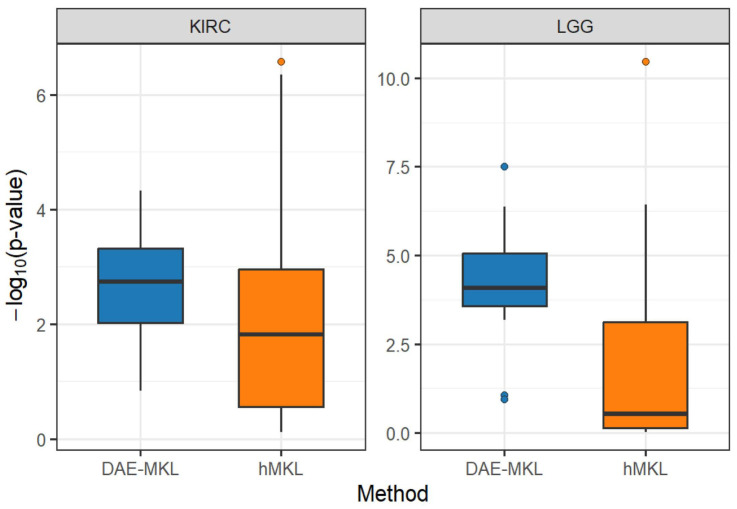
Distribution of −log_10_ (*p*-value) from log-rank tests across 20 random sample splits. Note: dots represent outliers.

We conducted a stability-based validation using random data splits to evaluate the robustness of subtyping results for both DAE-MKL and hMKL. Specifically, considering the sample sizes, we repeatedly sampled 70% of the patients from the KIRC dataset and 80% from the LGG dataset for training, and performed subtyping, repeating the procedure 20 times. The distribution of the log-rank test *p*-values across 20 runs is displayed in Figure 3. The mean p-values over 20 repetitions are summarized in Table 3, showing that DAE-MKL achieves smaller p-values and more consistent subtype identification compared to hMKL, suggesting superior robustness and clearer survival separation.

### 3.4. Analysis of KIRC Subtypes Identified by DAE-MKL

**Figure 4:**
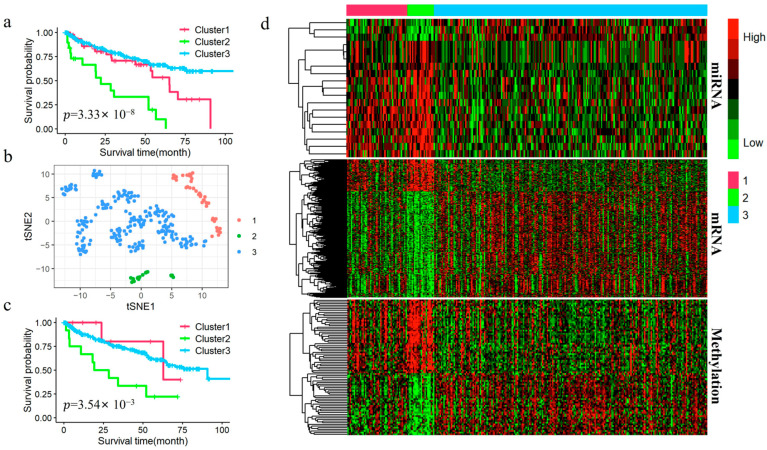
Clustering results for KIRC. (**a**) Kaplan–Meier survival curves for different subtypes identified by DAE-MKL. (**b**) t-SNE visualization of different subtypes identified by DAE-MKL. (**c**) Kaplan-Meier survival curves for different subtypes identified by hMKL. (**d**) Heatmaps of DEmiRNAs, DEmRNAs, and DMGs identified by DAE-MKL across different clusters, where each row represents an individual feature and each column corresponds to a patient. Red and green indicate relatively high and low expression levels, respectively.

To better understand molecular heterogeneity, we performed a subtype analysis of KIRC patients by integrating the three omics data types. We focused on our comparison with the original hMKL. Patients were classified into three subtypes based on the cost separation function and supported by findings from previous classical studies [7,15] (Figure 3). The baseline clinical data for the identified subtypes are presented in Table 4, which details demographic and clinical characteristics, including age, gender, pathologic stage, and survival status. As shown in Figure 4a,c, survival curves of the two methods suggest that the clusters obtained by DAE-MKL show more significant differences in overall survival with strong separation (log-rank *p*-value = 3.33 × 10^−8^) compared with the hMKL cluster result (log-rank *p*-value = 3.50 × 10^−3^). Furthermore, the t-SNE visualization showed that patient samples were clearly separated into distinct clusters by the DAE-MKL (Figure 4b). We further performed Cox regression analysis to evaluate the correlation between different subtypes and the survival outcome, adjusting for age, gender and pathological stage. As shown in Table 5, Cluster 2 had a 2.608-fold higher risk of death compared to Cluster 3 (p-value = 0.002), while patients in stage III and stage IV had a 3.922-fold and 9.334-fold higher risk of death, respectively, compared to those in stage I (which served as the reference group in the analysis) (p-value = 9.10 × 10^−5^ and 5.20 × 10^−12^).

### 3.5. Differential Expression Analysis for KIRC

**Figure 5:**
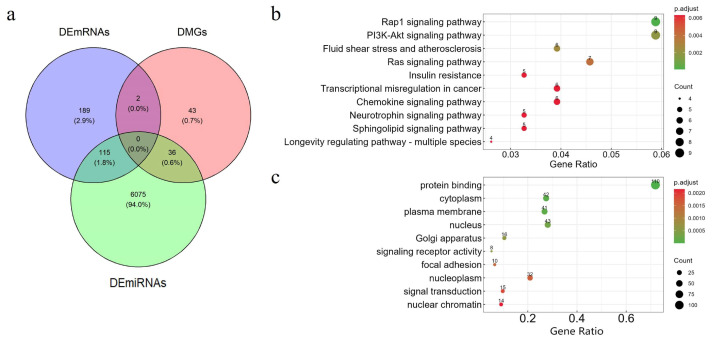
Functional annotation analysis of KIRC. (**a**) The number of differentially deregulated mRNAs, mRNAs targeted by miRNAs, and genes with DNA methylation across the three subtypes. (**b**) The top 10 enriched KEGG pathways of genes in KIRC. (**c**) The top 10 enriched GO terms of genes in KIRC.

We performed differential expression analysis between different subtypes in the KIRC dataset using the Kruskal–Wallis test and the hypergeometric distribution test, based on the subtypes identified by DAE-MKL. A total of 19 DEmiRNAs were identified, of which 10 were up-regulated and 9 were down-regulated; 306 DEmRNAs, of which 150 were up-regulated and 156 were down-regulated; 81 abnormal DNA methylation genes, among which 39 were hypermethylated and 42 were hypomethylated. The heatmap of differential expression in different omics data is shown in Figure 4d, which clearly highlights the significant heterogeneity between high- and low-risk KIRC patient groups across the three data types. Furthermore, using the miRWalk online tool to predict the target genes of DEmiRNAs, we identified 153 genes regulated by mRNA, miRNA, and DNA methylation. These genes are represented by the overlapping areas of the circles in the Venn diagram (Figure 5a).

### 3.6. Functional Annotation Analysis of Overlapping Genes in KIRC

We further performed functional annotation to explore the potential biological processes and pathways associated with the overlapping genes across the three omics datasets. The top 10 enriched KEGG pathways and GO biological processes with significant gene associations are shown in Figure 5b,c. GO analysis revealed that overlapping genes are primarily involved in the regulation of important biological processes, such as protein binding, cytoplasm, plasma membrane, and nucleus. Studies have suggested that the interaction between specific SARS-CoV-2 proteins and human mRNAs (SPBRs) may be implicated in the initiation and progression of KIRC. Targeting these binding proteins presents a potential novel therapeutic strategy for KIRC, offering a promising approach for anti-tumor treatment [43]. Additionally, the inhibition of signal transduction has emerged as a viable therapeutic avenue. Evidence indicates that signal transduction inhibitors, such as everolimus, when used in combination with other drugs, can reduce glucose and glutamine consumption, thereby exerting synergistic effects in countering the proliferation of renal cell carcinoma [44].

In addition, KEGG pathway analysis showed that these genes were significantly enriched in the Rap1 signaling pathway, the PI3K-Akt signaling pathway, the Ras signaling pathway and other tumor-associated signaling pathways. Evidence suggests that VEGF expression levels in tumor tissues can serve as a critical indicator of malignancy, invasiveness, and metastatic potential, making it a valuable prognostic marker for KIRC [45]. Notably, studies have shown that EGFR expression is closely associated with prognosis in patients with clear cell renal cell carcinoma [46].

### 3.7. Immune Cell Infiltration and Pathway Activity Analysis for KIRC

**Figure 6:**
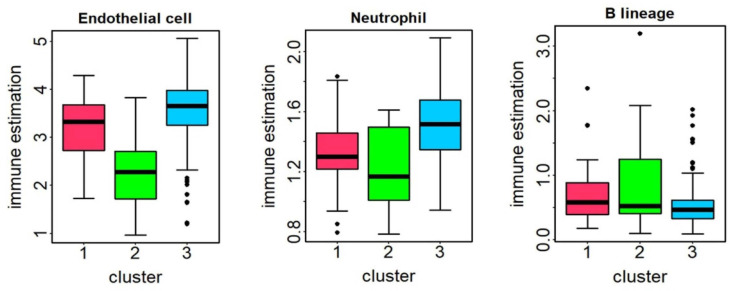
The difference in immune cell infiltration in different clusters of KIRC. The abundance of endothelial cells, neutrophils, and B-lineage in different clusters of KIRC. Note: Black dots represent outliers.

To investigate the association between different molecular subtypes and tumor-infiltrating immune cells as well as pathway activity, we conducted immune cell infiltration and pathway activity analyses on the KIRC dataset. As shown in Figure 6, three types of infiltrating cells including endothelial cells, neutrophils, and B-lineage, showed significant differences between the three subtypes. Cluster 3 had higher levels of endothelial cells and neutrophils, but lower levels of B-lineage compared to cluster 2, which was associated with a worse prognosis. We can observe that higher levels of endothelial cell infiltration are associated with better prognosis. This finding is consistent with the conclusion that increased endothelial cell infiltration is significantly linked to better outcomes in renal cell carcinoma [47]. Similarly, a study reported that KIRC patients with higher endothelial cell content generally experience better overall survival compared to those with lower endothelial cell content [48].

**Figure 7:**
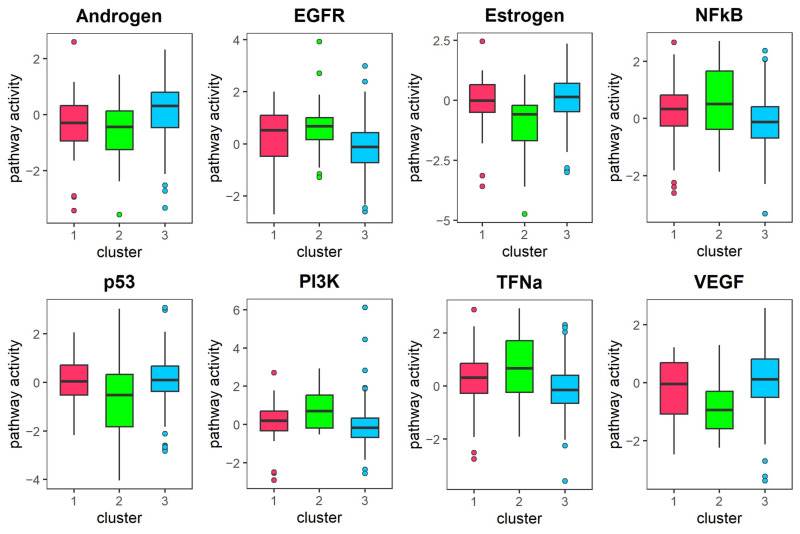
The difference in pathway activity in different clusters of KIRC. The pathway activity for Androgen, EGFR, Estrogen, NFk β, p53, PI3K, TNF α and VEGF in different clusters of KIRC. Note: dots represent outliers.

As shown in Figure 7, the eight most significant pathways are displayed. We can see that the activity of EGFR, NFk β, PI3K and TNF pathways in Cluster 2 with the worst overall survival is significantly higher than the other two clusters. Research has shown that NFk β is closely linked with key factors such as VEGF, EGFR, Bcl-2, and p53 in KIRC, and it represents a potential therapeutic target for overcoming chemotherapy resistance in this context. Increased NFk β activity is associated with elevated expression of Bcl-2, p53, VEGF, and EGFR [49]. In cancer cells, NFk β signaling contributes to processes such as cell proliferation, apoptosis regulation, angiogenesis, and chemo-radioresistance, while also holding significant diagnostic and prognostic value. Tumor-infiltrating macrophages secrete TNFα, which in turn promotes tumor growth. Interestingly, TNFα is also recognized as an antitumor cytokine due to its capacity to induce hemorrhagic necrosis within tumors. It influences both cancerous and normal cells, thereby playing a critical role in inflammation and immune surveillance. p53, a potent tumor suppressor, when mutated or inactivated, leads to abnormal proliferation and survival of renal clear cell carcinoma. Variations in pathway activity reflect, to some extent, the heterogeneity observed across different KIRC subtypes [50].

### 3.8. Analysis of LGG Subtypes Identified by DAE-MKL

**Figure 8:**
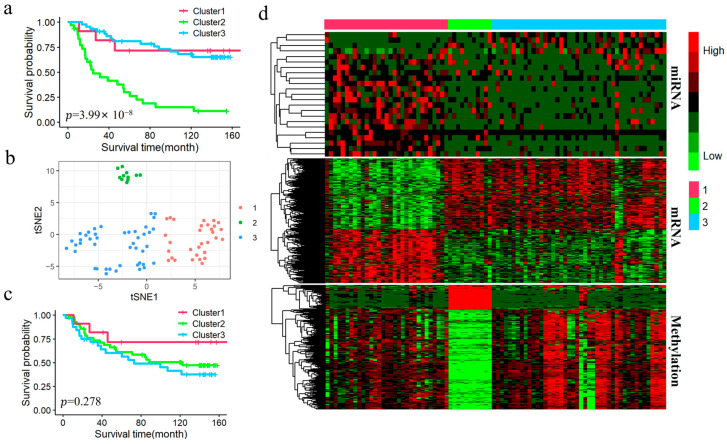
Clustering results of LGG. (**a**) Kaplan–Meier survival curves for different subtypes identified by DAE-MKL. (**b**) t-SNE visualization of different subtypes identified by DAE-MKL. (**c**) Kaplan–Meier survival curves for different subtypes identified by hMKL. (**d**) Heatmaps of DEmiRNAs, DEmRNAs, and DMGs identified by DAE-MKL across different clusters, where each row represents an individual feature and each column corresponds to a patient. Red and green indicate relatively high and low expression levels, respectively.

We implemented similar analysis strategies for the LGG data, patients were classified into three subtypes based on the cost separation function. The baseline clinical data for the identified subtypes are presented in Table 6, which details demographic and clinical characteristics, including age, gender, pathologic stage, and survival status. As shown in Figure 8a,c, survival curves of the two methods suggest that the clusters obtained by DAE-MKL show more significant differences in overall survival with strong separation (log-rank *p*-value = 3.99× 10^−8^) compared with the hMKL cluster result (log-rank *p*-value = 0.278). In addition, the t-SNE visualization indicated that the DAE-MKL effectively separated patient samples into well-defined clusters (Figure 8b). We further performed Cox regression analysis to evaluate the correlation between different subtypes and the survival outcome, adjusting for age, gender and pathological stage. As shown in Table 7, Cluster 1 had a 3.568-fold higher risk of death compared to Cluster 3 (p-value = 0.017).

### 3.9. Differential Expression Analysis for LGG

**Figure 9:**
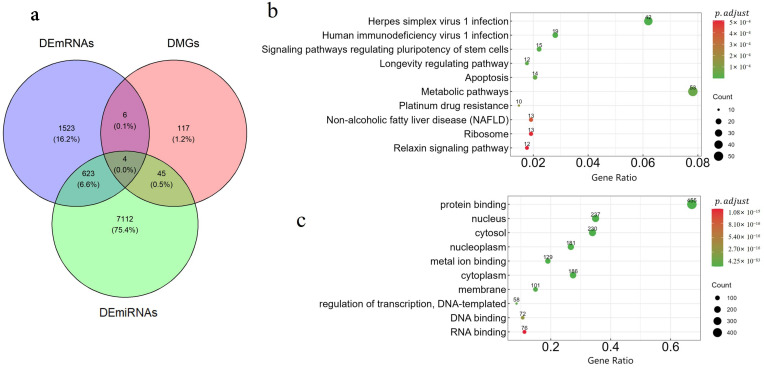
Functional annotation analysis of LGG. (**a**) The number of differentially deregulated mRNAs, mRNAs targeted by miRNAs, and genes with DNA methylation across the three subtypes. (**b**) The top 10 enriched KEGG pathways of genes in LGG. (**c**) The top 10 enriched GO terms of genes in LGG.

We identified a total of 23 DEmiRNAs, all of which were up-regulated; 2156 DEmRNAs, of which 912 were up-regulated and 1244 were down-regulated; 172 abnormal DNA methylation genes, among which 12 were hypermethylated and 172 were hypomethylated. The heatmap of differential expression across the three omics data types is presented in Figure 8d, clearly illustrating the significant heterogeneity between high- and low-risk KIRC patient groups. Additionally, using the miRWalk online tool to predict the target genes of the DEmiRNAs, we identified 627 genes regulated by mRNA, miRNA, and DNA methylation, as depicted by the overlapping areas of the circles in the Venn diagram (Figure 9a).

### 3.10. Functional Annotation Analysis of Overlapping Genes in LGG

We conducted functional annotation analyses to delve deeper into the biological significance of the overlapping genes identified across the three omics datasets. The results highlight the top 10 enriched KEGG pathways and GO biological processes that exhibit significant associations with these genes, as illustrated in Figure 9b,c. GO term analysis showed that the genes were primarily involved in maintaining cell function and regulating vital activities, and played a role in essential biological processes such as protein synthesis, and cellular communication, gene expression. KEGG pathway analysis showed that the genes were mainly enriched in the Human Immunodeficiency Virus 1 infection pathway, apoptosis pathway, and metabolic pathways. Evidence showed that low-grade glioma was significantly associated with improved OS in HIV-infected patients with glioma [51]. Notably, the up-regulated expression of *HMGB1* may play key roles in the occurrence, development, invasion and metastasis of gliomas. The inhibition of *HMGB1* gene expression may inhibit the growth and proliferation of glioma cells and promote apoptosis; overexpression of *HMGB1* may promote the growth and proliferation of glioma cells and inhibit apoptosis [52]. Studies have demonstrated that metabolic alterations may promote tumor cell proliferation and migration [53].

### 3.11. Immune Cell Infiltration and Pathway Activity Analysis for LGG

**Figure 10:**
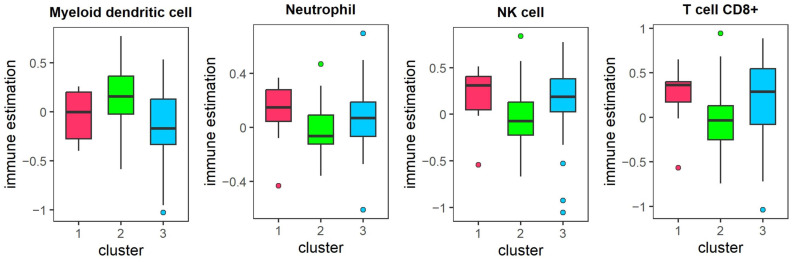
The difference of immune cell infiltration in different clusters of LGG. The abundance of myeloid dendritic cell, neutrophils, NK cell and CD8+ T cells in different clusters of LGG. Note: dots represent outliers.

As shown in Figure 10, immune infiltration analysis revealed significant variations in multiple immune cell types, including myeloid dendritic cells, neutrophils, natural killer cells and CD8+ T cells. Notably, neutrophils, NK cells, and CD8+ T cells exhibited significantly higher abundance in cluster 1, whereas myeloid dendritic cells were abundant in cluster 2. Current research indicates that the composition of tumor-infiltrating immune cells is closely related to survival outcomes across various cancer types. Specifically, neutrophil infiltration has been associated with a better prognosis in multiple malignancies and can be therapeutically activated to enhance tumor-killing effects [54]. Moreover, studies suggest that as tumor grade progresses, the proportions of both CD8+ and CD4+ tumor-infiltrating T cells tend to increase. Glioma patients with higher CD8+ T cell counts at diagnosis consistently demonstrate improved survival outcomes compared to those with lower levels [55].

**Figure 11:**
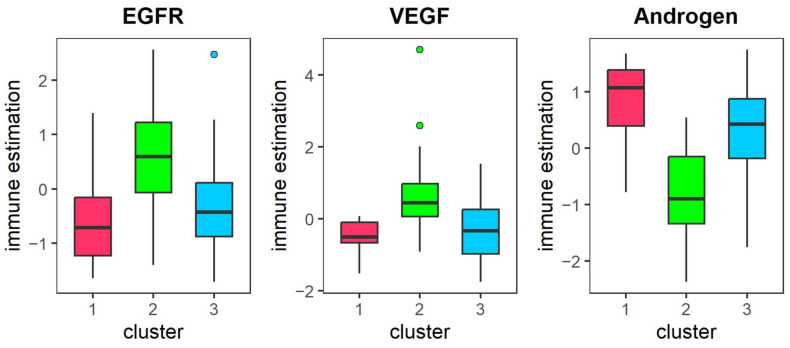
The difference of pathway activity in different clusters of LGG. The pathway activity for EGFR, VEGF and Androgen in different clusters of LGG. Note: dots represent outliers.

Figure 11 displays the three pathway activities with significant differences between the three clusters (p < 0.05), with Cluster 1 showing the highest activity in the Androgen pathways and the lowest activity in the EGFR and VEGF pathways. Androgen, a steroid hormone, binds to the androgen receptor (AR), liberating it from heat shock proteins and facilitating its translocation into the nucleus. This nuclear translocation impacts the expression of certain genes, ultimately promoting glioma cell proliferation [56,57]. The EGFR pathway is crucial for the secretion of several cytokines and the infiltration of immune cells. Activation of the EGFR signaling pathway can induce the expression of *CCL2*, suggesting that targeting EGFR may offer therapeutic benefits [58]. Additionally, miR-376a directly regulates the expression of *SIRT1* in glioma cells, thereby suppressing the VEGF signaling pathway and ultimately inhibiting glioma cell proliferation. Therefore, inhibiting the activities of the EGFR and VEGF pathways may contribute to a better prognosis for patients in cluster 1 [59].

## 4. Discussion

Cancer is a highly heterogeneous disease with unique genomic and phenotypic features, as well as intricate molecular alterations. The identification of distinct subtypes is crucial for uncovering potential therapeutic targets and facilitating precision medicine. While recent multi-omics data integration methods have improved subtype classification, they often fail to effectively extract the most informative features from high-dimensional data for practical applications. To address this limitation, we propose a novel deep learning-based framework, DAE-MKL, which integrates denoising autoencoders to reduce noise in multi-omics data, thereby enhancing cancer subtyping. The effectiveness and advantages of our method are demonstrated through simulations and applications to two cancer datasets, showing that DAE-MKL identifies molecular subtypes with greater robustness and efficiency compared to state-of-the-art approaches.

The main contribution of this approach can be summarized as follows. First, autoencoders are widely recognized as powerful unsupervised deep learning models, extensively used for constructing lower-dimensional representations for multi-omics data integration. In this study, we utilized denoising autoencoders for both dimensionality reduction and feature extraction, effectively eliminating irrelevant information and enhancing data quality. Second, the lower-dimensional representations generated by the autoencoder were input into a hierarchical Multiple Kernel Learning framework. Within this framework, a composite kernel is learned for each omics type by optimizing the corresponding kernel parameters, and all composite kernels are subsequently combined through a weighted linear combination to form a unified fused kernel. This approach enhances the accuracy and robustness of cancer subtype identification based on heterogeneous multi-omics data.

We demonstrate the benefits of DAE-MKL through extensive simulations and applications to two cancer types from TCGA and CGGA datasets (KIRC and LGG). The simulation results show that the NMI of different methods increases with the signal ratio and decreases with the noise level. DAE-MKL outperforms SAE-MKL, hMKL, CIMLR, SNF and AE-MKL across all settings under the two simulation scenarios. In real-data analyses, DAE-MKL successfully identified distinct subtypes with significant differences in survival outcomes and potential cancer-associated biomarkers. The discovered subtypes show significant differences in gene enrichment, functional enrichment, cancer-related biological pathways, and immune cell infiltration. These findings further highlight the advantages of the DAE-MKL approach in integrative subtyping. By providing a holistic perspective that reveals biological mechanisms across various regulatory layers, DAE-MKL enables the identification of molecular subtypes and underscores its potential for uncovering the underlying disease mechanisms.

Clinically, KIRC is categorized into three distinct subtypes with strong separation in survival curves. Specifically, patients in Cluster 2 exhibited a 2.608-fold higher risk of death compared to those in Cluster 3. Similarly, patients in stages III and IV had a 3.922-fold and 9.334-fold higher risk of death, respectively, relative to those in stage I. These findings underscore the prognostic significance of molecular subtyping in KIRC. To further clarify the biological underpinnings of these subtypes, we conducted a comprehensive analysis, highlighting their potential clinical relevance. In the differential analysis, *SEMA3C* was found to be upregulated. As a Class-3 semaphorin, it has been implicated in immune regulation, angiogenesis, and tumor progression, and its elevated expression is linked to poorer survival outcomes [60]. Notably, three types of infiltrating cells—endothelial cells, neutrophils, and B-lineage cells—exhibited significant differences between the three subtypes. Additionally, the activities of the EGFR, NFkβ, PI3K and TNFα pathways were significantly higher in Cluster 2, which had the worst overall survival, compared to the other two clusters. In KIRC, EGFR overexpression is recognized as a crucial factor contributing to the initiation and progression of renal cell carcinoma [61]. Functional enrichment analysis revealed a notable enrichment of these genes in specific GO terms, highlighting their involvement in key biological processes. Additionally, KEGG pathway analysis demonstrated that these genes play a pivotal role in several critical pathways, underscoring their importance in cellular functions.

For LGG, the DAE-MKL approach identified three distinct molecular subtypes, with patients in Cluster 1 exhibiting a 3.568-fold higher risk of death compared to those in Cluster 3. Similar to KIRC, downstream analyses were conducted based on these molecular subtypes. In the differential analysis, *PCDH15* was found to be uniquely overexpressed in low-grade glioma, and previous studies have demonstrated that its aberrant expression profoundly affects patient prognosis [62]. Moreover, members of the transient receptor potential vanilloid (TRPV) channel family, particularly *TRPV6*, have been reported to play crucial roles in the development and progression of LGG [63]. We observed significant differences in two immune cell infiltration patterns and three pathway activities across the subtypes. GO term analysis highlighted that the genes were primarily involved in maintaining cellular functions and regulating essential biological processes. KEGG pathway analysis demonstrated that the genes were predominantly enriched in the Human Immunodeficiency Virus 1 (HIV-1) infection pathway, apoptosis pathway, and metabolic pathways. These findings underscore the importance of integrative molecular subtyping in advancing precision medicine approaches for both KIRC and LGG.

Despite the promising results of this study, several limitations should be acknowledged. First, our current approach focused exclusively on autoencoders architectures; future work will explore a broader range of deep learning methods based on dimensionality reduction to enhance model performance and interpretability. Second, although bioinformatics analysis identified several cancer-related biomarkers, the underlying causal mechanisms of these biomarkers remain unclear and require further experimental validation in biological studies. Moreover, exploring the influence of kernel selection on model performance could provide deeper insights into the flexibility and adaptability of MKL methods for the specific nature of input datasets.

In conclusion, our proposed DAE-MKL framework effectively reduces noise and extracts robust feature representations from high-dimensional data, achieving superior model performance, improving the understanding of complex diseases, and enabling personalized treatment strategies.

## Figures and Tables

**Table 1:** Performance measured by NMI in simulation Scenario I.

	Sign%	Method	Low-Noise	Medium-Noise	High-Noise
SimData1	5%	**DAE-MKL**	**0.883 (0.036)**	**0.601 (0.053)**	**0.412 (0.044)**
		SAE-MKL	0.863 (0.038)	0.562 (0.050)	0.387 (0.047)
		AE-MKL	0.170 (0.200)	0.156 (0.207)	0.111 (0.141)
		hMKL	0.583 (0.049)	0.311 (0.035)	0.161 (0.046)
		CIMLR	0.372 (0.042)	0.253 (0.047)	0.142 (0.044)
		SNF	0.339 (0.031)	0.262 (0.030)	0.192 (0.035)
	7.5%	**DAE-MKL**	**0.962 (0.024)**	**0.816 (0.041)**	**0.628 (0.051)**
		SAE-MKL	0.953 (0.031)	0.801 (0.047)	0.600 (0.052)
		AE-MKL	0.381 (0.208)	0.232 (0.208)	0.244 (0.225)
		hMKL	0.722 (0.030)	0.503 (0.051)	0.332 (0.040)
		CIMLR	0.540 (0.059)	0.344 (0.038)	0.264 (0.043)
		SNF	0.417 (0.042)	0.316 (0.033)	0.269 (0.030)
	10%	**DAE-MKL**	**0.979 (0.039)**	**0.899 (0.038)**	**0.780 (0.048)**
		SAE-MKL	0.934 (0.129)	0.893 (0.032)	0.769 (0.048)
		AE-MKL	0.328 (0.194)	0.209 (0.200)	0.206 (0.244)
		hMKL	0.774 (0.028)	0.626 (0.045)	0.468 (0.043)
		CIMLR	0.695 (0.052)	0.408 (0.049)	0.328 (0.041)
		SNF	0.554 (0.061)	0.356 (0.031)	0.313 (0.030)
SimData2	5%	**DAE-MKL**	**0.818 (0.055)**	**0.623 (0.050)**	**0.412 (0.060)**
		SAE-MKL	0.800 (0.052)	0.582 (0.056)	0.391 (0.060)
		AE-MKL	0.218 (0.225)	0.254 (0.219)	0.160 (0.152)
		hMKL	0.489 (0.056)	0.304 (0.037)	0.155 (0.052)
		CIMLR	0.407 (0.032)	0.265 (0.048)	0.135 (0.049)
		SNF	0.371 (0.033)	0.275 (0.039)	0.189 (0.038)
	7.5%	**DAE-MKL**	**0.909 (0.040)**	**0.756 (0.064)**	**0.637 (0.051)**
		SAE-MKL	0.894 (0.066)	0.740 (0.065)	0.603 (0.057)
		AE-MKL	0.304 (0.175)	0.244 (0.226)	0.285 (0.218)
		hMKL	0.599 (0.077)	0.447 (0.046)	0.319 (0.038)
		CIMLR	0.516 (0.040)	0.368 (0.042)	0.277 (0.047)
		SNF	0.446 (0.045)	0.342 (0.039)	0.280 (0.038)
	10%	**DAE-MKL**	**0.929 (0.042)**	0.827 (0.054)	**0.720 (0.073)**
		SAE-MKL	0.870 (0.127)	0.830 (0.043)	0.716 (0.056)
		AE-MKL	0.304 (0.157)	0.200 (0.187)	0.198 (0.225)
		hMKL	0.727 (0.070)	0.534 (0.066)	0.422 (0.044)
		CIMLR	0.631 (0.049)	0.440 (0.033)	0.353 (0.041)
		SNF	0.534 (0.044)	0.388 (0.040)	0.330 (0.037)

Note: The NMI values are presented as mean and standard deviation of 1000 simulation results. The method(s) with the best performance is(are) highlighted in bold fonts at a given noise level.

**Table 2:** Comparison of subtyping results of different integration methods.

Cancer	DAE-MKL	SAE-MKL	AE-MKL	hMKL	CIMLR	SNF
KIRC	**3 (3.33 × 10^−8^)** ^a^	3 (4.08 × 10^−6^)	2 (0.41)	3 (3.50 × 10^−3^)	2 (4.11 × 10^−8^)	4 (0.02)
LGG	**3 (3.99 × 10^−8^)**	3 (3.99 × 10^−8^)	5 (1.42 × 10^−6^)	3 (0.278)	3 (0.267)	3 (1.90 × 10^−6^)

^a^ The number of optimal subtypes identified by each method is listed, with the corresponding log-rank test p-value shown in parentheses. For DAE-MKL, SAE-MKL, AE-MKL, hMKL and CIMLR, the number of subtypes was determined based on the separation cost. SNF selected the optimal number using the eigenvalue gaps [42].

**Table 3:** The mean p-value of the log-rank test over 20 random sample splits.

Method	Mean p-Value of the Log-Rank Tests	
	KIRC	LGG
DAE-MKL	**3 (0.018)** ^a^	**3 (0.010)**
hMKL	3 (0.162)	3 (0.351)

^a^ The numbers of optimal subtypes identified by the DAE-MKL and hMKL methods are listed, with the corresponding mean log-rank test p-values over 20 random sample splits shown in parentheses.

**Table 4:** Baseline clinical data for different subtypes of KIRC patients.

Items	Cluster 1	Cluster 2	Cluster 3
Number of patients (n,%)	48 (16.84)	21 (7.37)	216 (75.79)
Age (mean ± sd)	58.67 ± 12.69	64.19 ± 9.00	59.87 ± 10.15
Gender (n,%)			
Male	15 (31.25)	8 (38.10)	77 (35.65)
Female	33 (68.75)	13 (61.90)	139 (64.35)
Pathologic stage (n,%)			
I	20 (41.67)	4 (19.05)	114 (52.78)
II	4 (8.33)	1 (4.76)	24 (11.11)
III	12 (25.00)	8(38.09)	44 (20.37)
IV	12 (25.00)	8 (38.10)	34 (15.74)
Survival status (n,%)			
Survival	30 (62.50)	6 (28.57)	167 (77.31)
Death	18 (37.50)	15 (71.43)	49 (22.69)

Note: Categorical variables are presented as counts and percentages, while continuous variables are presented as mean ± standard deviation ($\bar{x}\pm s$).

**Table 5:** Cox regression analysis of 285 KIRC patients.

Items	b (S.E)	Z	p	HR (95% CI)
Subtypes				
Cluster 1	0.232 (0.282)	0.823	0.410	1.261 (0.726–2.193)
Cluster 2 *	0.959 (0.310)	3.091	**0.002**	2.608 (1.420–4.790)
Age	0.015 (0.013)	1.195	0.232	1.015 (0.991–1.040)
Gender	−0.108 (0.242)	−0.445	0.656	0.898 (0.559–1.442)
Pathologic stage				
II	0.494 (0.528)	0.935	0.350	1.638 (0.582–4.610)
III *	1.367 (0.349)	3.915	**9.1 × 10^−5^**	3.922 (1.979–7.776)
IV *	2.234 (0.324)	6.901	**5.2 × 10^−12^**	9.334 (4.949–17.601)

* Shows statistically significant (*p* < 0.05); Cluster 3 served as the reference for comparing distinctions among subtypes, while Stage I was employed as the reference for the comparison of differences across pathological stages; HR = Hazard Ratio.

**Table 6:** Baseline clinical data for different subtypes of LGG patients.

Items	Cluster 1	Cluster 2	Cluster 3
Number of patients (n,%)	31 (36.05)	11 (12.79)	44 (51.16)
Age (mean ± sd)	41.77 ± 14.38	38.36 ± 9.15	36.34 ± 9.48
Gender (n,%)			
Male	16 (51.61)	5 (45.45)	25 (56.82)
Female	15 (48.39)	6 (54.55)	19 (43.18)
Pathologic stage (n,%)			
II	3 (9.68)	10 (90.91)	39 (88.64)
III	28 (90.32)	1 (9.09)	5 (11.36)
Survival status (n,%)			
Survival	6 (19.35)	8 (72.73)	30 (68.18)
Death	25 (80.65)	3 (27.27)	14 (31.82)

Note: Categorical variables are presented as counts and percentages, while continuous variables are presented as mean ± standard deviation ($\bar{x}\pm s$).

**Table 7:** Cox regression analysis of 86 LGG patients.

Items	*b* (*S.E*)	*Z*	*p*	*HR* (95% *CI*)
Subtype				
Cluster1 *	1.272 (0.531)	2.394	**0.017**	3.568 (1.259–10.112)
Cluster 2	−0.076 (0.641)	−0.118	0.906	0.927 (0.264–3.259)
Age	0.019 (0.012)	1.605	0.109	1.020 (0.996–1.044)
Gender	0.060 (0.318)	0.187	0.851	1.061 (0.569–1.981)
Pathologic stage				
III	0.603 (0.472)	1.277	0.202	1.828 (0.724–4.613)

* Shows statistically significant (*p* < 0.05); Cluster 3 served as the reference for comparing distinctions among subtypes, while Stage II was employed as the reference for the comparison of differences across pathological stages; HR = Hazard Ratio.

## Data Availability

The KIRC and LGG datasets, along with the source code of DAE-MKL used in this study, have been deposited in an online repository and are available at https://github.com/biostatYao/DAE-MKL (accessed 18 October 2025). All data generated or analyzed during this study are included in this article and its Supplementary Materials. Further inquiries can be directed to the corresponding authors.

## Supplementary Materials

The following supporting information can be downloaded at: https://www.mdpi.com/article/10.3390/genes16111246/s1, Table S1. Summarizes the detailed NMI values; Figure S1. Sensitivity analysis of input-layer and hidden-layer dropout ratios in the DAE-MKL; Table S2. Performance measured by NMI in simulation Scenario II; Figure S2. Distribution of NMI values under Scenario Ⅱ. a. NMI value distribution for SimData1. b. NMI value distribution for SimData2; Table S3. Internal validation indices for DAE-MKL identified subtypes in KIRC and LGG.
