# Supplementary material for: Multi-Omics Data Integration for Improved Cancer Subtyping via Denoising Autoencoder-Based Multi-Kernel Learning
Source: Genes (Basel). 2025 Oct 22;16(11):1246. doi: 10.3390/genes16111246 (PMC12652543; doi:10.3390/genes16111246)
Supplement: Supplementary file 1 [file genes-16-01246-s001.zip › genes-3927892-supplementary.pdf]

Supplemental materials for "**Multi-Omics Data Integration for Improved Cancer Subtyping via Denoising Autoencoder-Based Multi-Kernel Learning**"

Xiukun Yao [1,2,3], Tong Wang [4,5], Qi Yang [4,5], Jiawen Wang [1,2,3], Yao Qi [1,2,3], Tong Xu [4,5], Zhiwen Wei [1,2,3], Yuehua Cui [6], Hongyan Cao [4,5] and Keming Yun [1,2,3,*]

1 Academy of Forensic Medicine, Shanxi Medical University, Jinzhong 030600, China

2 Key Laboratory of Forensic Medicine in Shanxi Province, Jinzhong 030600, China

3 Key Laboratory of Forensic Toxicology, Ministry of Public Security, Jinzhong 030600, China

4 Shanxi Provincial Key Laboratory of Major Diseases Risk Assessment, Department of Health Statistics, School of Public Health, Shanxi Medical University, Jinzhong 030600, China

5 MOE Key Laboratory of Coal Environmental Pathogenicity and Prevention, Shanxi Medical University, Jinzhong 030600, China

6 Department of Statistics and Probability, Michigan State University, East Lansing, MI 48824, USA

* Correspondence: yunkeming@sxmu.edu.cn

1. Supplementary Note S1: Sensitivity analysis of dropout ratios in DAE-MKL

For a detailed evaluation of the dropout parameters, we systematically varied the input-layer dropout (0.1-0.9) and hidden-layer dropout (0.3-0.9) using simulated datasets with variance = 12 and signal-to-noise ratio = 10%. The normalized mutual information (NMI) values obtained by the DAE-MKL method are summarized in Table S1. To provide a more intuitive visualization, Figure S1 shows NMI as a function of input-layer dropout for different hidden-layer dropout values. These results indicate that the DAE-MKL model achieves the best clustering performance when the input-layer dropout ratio is between 0.1-0.3 and the hidden-layer dropout ratio is between 0.3-0.5, which justifies our choice of these parameters in all subsequent analyses.

Table S1. Summarizes the detailed NMI values

ID	HD=0.3	HD=0.5	HD=0.7	HD=0.9
0.1	0.779	0.730	0.648	0.223
0.2	0.789	0.713	0.611	0.204
0.3	0.778	0.694	0.588	0.196
0.4	0.733	0.687	0.533	0.148
0.5	0.729	0.637	0.495	0.125
0.6	0.625	0.554	0.436	0.098
0.7	0.481	0.454	0.324	0.056
0.8	0.303	0.276	0.162	0.046
0.9	0.075	0.046	0.038	0.045

Note: ID = input dropout; HD = hidden dropout.

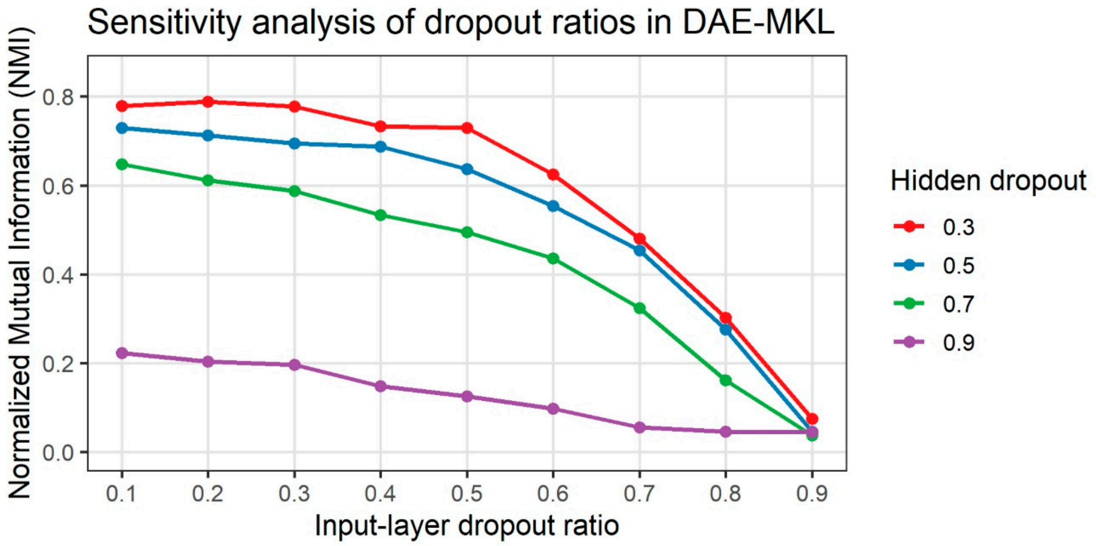

Figure S1. Sensitivity analysis of input-layer and hidden-layer dropout ratios in the DAE-MKL

2. Supplementary Note S2: Results of Simulation Scenario II

Table S2 and Figure S2 present the NMI values of DAE-MKL, SAE-MKL, AE-MKL, hMKL, CIMLR, and SNF across 1000 replicates under Scenario II. Similar to Scenario I, all methods show increasing NMI values as the signal ratio increases when the noise ratio is held constant. DAE-MKL tends to achieve higher NMI values than the other methods under most experimental conditions, indicating superior performance in subtype identification and a better ability to capture the clustering structure of multi-omics data. Under a 10% signal strength with high noise, for instance, DAE-MKL attains an NMI of 0.776, whereas SAE-MKL, AE-MKL, hMKL, CIMLR, and SNF reach 0.744, 0.365, 0.416, 0.398, and 0.367, respectively. Overall, although SimData2, which simulates fuzzy boundaries, shows a slight decrease in NMI across all methods, the relative performance trend remains consistent. These results corroborate the findings from the main simulation study and demonstrate the robustness of DAE-MKL in various settings.

Table S2. Performance measured by NMI in simulation Scenario II

	Sign%	Method	Low-noise	Medium-noise	High-noise
SimData1	5%	**DAE-MKL**	**0.844(0.078)**	**0.621(0.068)**	**0.473(0.049)**
		SAE-MKL	0.824(0.069)	0.566(0.055)	0.446(0.040)
		AE-MKL	0.218(0.231)	0.230(0.237)	0.189(0.192)
		hMKL	0.435(0.027)	0.361(0.033)	0.245(0.049)
		CIMLR	0.423(0.029)	0.310(0.045)	0.189(0.053)
		SNF	0.399(0.034)	0.306(0.033)	0.221(0.037)
	7.5%	**DAE-MKL**	0.906(0.072)	**0.786(0.089)**	**0.645(0.079)**

		SAE-MKL	0.914(0.079)	0.756(0.088)	0.604(0.070)
		AE-MKL	0.234(0.168)	0.332(0.265)	0.357(0.248)
		hMKL	0.447(0.029)	0.423(0.024)	0.381(0.025)
		CIMLR	0.487(0.040)	0.403(0.028)	0.335(0.049)
		SNF	0.453(0.042)	0.373(0.033)	0.322(0.037)
	10%	**DAE-MKL**	0.920(0.095)	0.856(0.078)	**0.776(0.085)**
		SAE-MKL	0.922(0.124)	0.869(0.058)	0.744(0.086)
		AE-MKL	0.292(0.131)	0.261(0.237)	0.365(0.267)
		hMKL	0.510(0.075)	0.441(0.030)	0.416(0.022)
		CIMLR	0.590(0.053)	0.442(0.026)	0.398(0.035)
		SNF	0.526(0.039)	0.417(0.032)	0.367(0.034)
SimData2	5%	**DAE-MKL**	**0.757(0.085)**	**0.549(0.059)**	**0.448(0.047)**
		SAE-MKL	0.711(0.084)	0.507(0.042)	0.417(0.041)
		AE-MKL	0.150(0.185)	0.169(0.206)	0.159(0.169)
		hMKL	0.413(0.020)	0.347(0.030)	0.237(0.044)
		CIMLR	0.406(0.024)	0.294(0.047)	0.169(0.057)
		SNF	0.386(0.033)	0.299(0.036)	0.213(0.037)
	7.5%	**DAE-MKL**	0.829(0.082)	**0.686(0.089)**	**0.573(0.060)**
		SAE-MKL	0.834(0.084)	0.649(0.082)	0.537(0.050)
		AE-MKL	0.310(0.215)	0.318(0.245)	0.377(0.200)
		hMKL	0.439(0.027)	0.408(0.024)	0.369(0.028)
		CIMLR	0.455(0.031)	0.391(0.032)	0.318(0.046)
		SNF	0.428(0.039)	0.365(0.033)	0.314(0.037)
	10%	**DAE-MKL**	0.842(0.091)	0.763(0.089)	**0.664(0.083)**
		SAE-MKL	0.852(0.113)	0.766(0.092)	0.632(0.080)
		AE-MKL	0.306(0.150)	0.285(0.227)	0.386(0.238)
		hMKL	0.463(0.038)	0.425(0.024)	0.401(0.022)
		CIMLR	0.529(0.050)	0.430(0.032)	0.385(0.029)
		SNF	0.489(0.040)	0.398(0.032)	0.353(0.030)

Note: The NMI values are presented as mean and standard deviation of 1000 simulation results. The method(s) with the best performance is(are) highlighted in bold fonts at a given noise level.

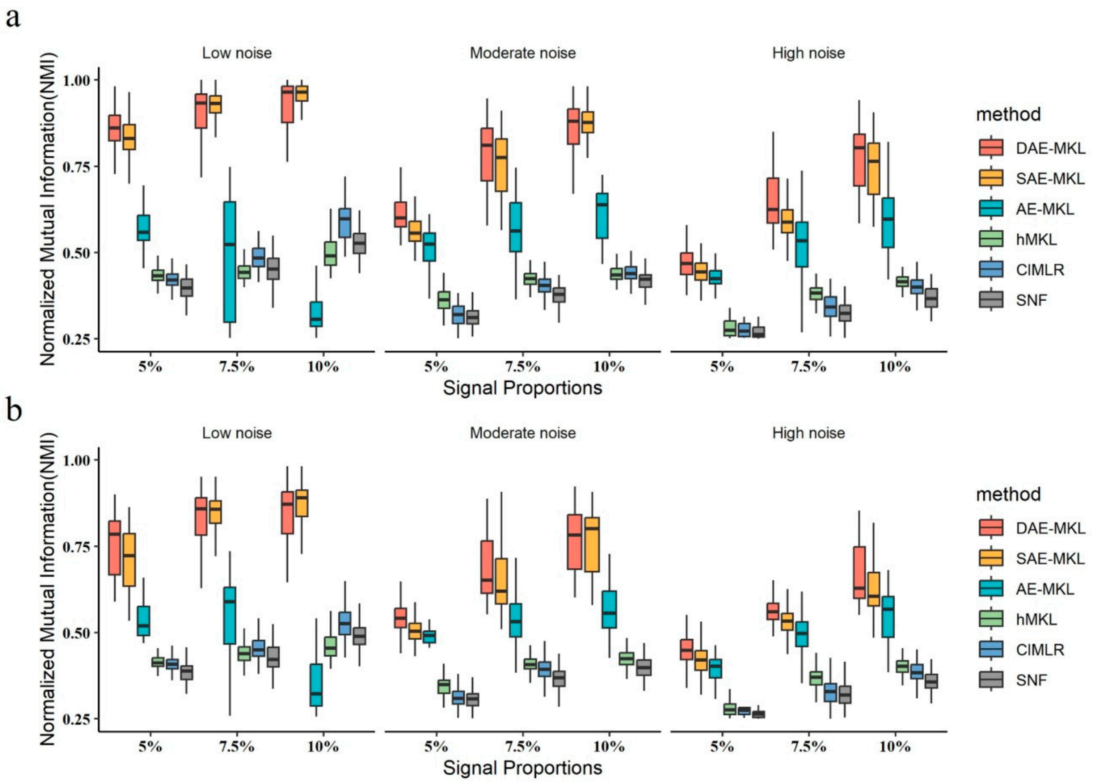

Figure S2. Distribution of NMI values under Scenario II. a. NMI value distribution for SimData1. b. NMI value distribution for SimData2.

3. Supplementary Note S3: Internal validation of DAE-MKL clustering results

To enhance the internal validation of our proposed method, we computed several internal clustering validation indices-Connectivity, Silhouette Width, and Dunn Index-for the three identified subtypes in both the KIRC and LGG datasets. The connectivity indicates the degree of connectedness of the clusters, as determined by the k-nearest neighbors. The connectivity has a value between 0 and infinity and should be minimized. Both the Silhouette Width and the Dunn Index combine measures of compactness and separation of the clusters. The Silhouette Width is the average of each observation's Silhouette value. The Silhouette value measures the degree of confidence in a particular clustering assignment and lies in the interval [-1,1], with well-clustered observations having values near 1 and poorly clustered observations having values near -1. The results are summarized in Table S3, showing that the identified clusters exhibit reasonable compactness and separation.

Table S3. Internal validation indices for DAE-MKL identified subtypes in KIRC and LGG

Dataset	Connectivity	Dunn	Silhouette
KIRC	6.76	0.52	0.33

LGG	14.07	0.43	0.21
